# High-affinity human PD-L1 variants attenuate the suppression of T cell activation

**DOI:** 10.18632/oncotarget.21729

**Published:** 2017-10-10

**Authors:** Zhaoduan Liang, Ye Tian, Wenxuan Cai, Zhiming Weng, Yanyan Li, Huanling Zhang, Yifeng Bao, Yi Li

**Affiliations:** ^1^ State Key Laboratory of Respiratory Disease, Guangzhou Institutes of Biomedicine and Health, Chinese Academy of Sciences, Guangzhou, China; ^2^ School of Life Sciences, University of Science and Technology of China, Hefei, China; ^3^ XiangXue Life Sciences Research Center, XiangXue Pharmaceutical Co. Ltd., Guangzhou, China

**Keywords:** high affinity, programmed cell death protein 1 (PD-1), PD-ligand 1 (PD-L1), soluble PD-L1, T cell, Immunology and Microbiology Section, Immune response, Immunity

## Abstract

The activated T cells can be suppressed by programed death-1 (PD-1) axis through low affinity interaction between PD-1 and PD-ligand 1 (PD-L1) in solution or on antigen presenting cells. In clinic, the concentration of soluble PD-L1 in peripheral blood negatively correlates with cancer prognosis. However, there is little information about the relation between the affinity of PD-1/PD-L1 interaction and the suppressive capacity of PD-1 axis. In this study, we analyzed inhibitory roles of high affinity soluble human PD-L1 (hPD-L1) variants, which were generated with directed molecular evolution. Resultant two clones L3C7-hPD-L1 and L3B3-hPD-L1 showed over 20 folds greater affinity than that of native hPD-L1. We found that L3B3-hPD-L1 and L3C7-hPD-L1 could compete with an anti-PD-1 antibody (EH12.1) for binding to hPD-1. More importantly, although native soluble hPD-L1 can induce suppressive effects on activated T cells, we found L3B3-hPD-L1 and L3C7-hPD-L1 attenuated the strength of PD-1 axis for suppressing the proliferation and interferon γ (IFN-γ) secretion of PBMC. In conclusion, our data provide direct evidence in which immune checkpoint receptor-ligand interactive strength can alter the the suppressive function, in particular, the suppressive capacity of PD-1 axis could be decreased with enhanced affinity of soluble PD-L1 and PD-1 interaction. Our study might provide a new direction for manipulating immune checkpoints.

## INTRODUCTION

As retaining the function of induced long-lasting immune responses, particularly against infectious diseases and tumor, T cells are the essential and critical component in adaptive immunity. Once activated, however, T cells begin to express other receptors, which include those receptors of negative co-stimulatory molecules such as programmed cell death protein 1 (PD-1) that suppresses the immune response [1]. PD-1 is encoded by the *PDCD1* gene in human, which was upregulated in a T-cell hybridoma undergoing apoptotic cell death [2, 3]. Over expression of PD-1 has related to a broad range of activated cells, including T cells [4-6]. PD-1 is a type I transmembrane monomeric protein, has a cytoplasmic tail containing one immune receptor tyrosine-based inhibition motif (ITIM) and one immune receptor tyrosine-based switch motif (ITSM), which play a fundamental role in the PD-1 suppressive function [7]. Two PD-1 ligands have been identified as programmed cell death ligand 1 (PD-L1) and PD-L2 [3, 8-10]. PD-L1 is distributed broadly in normal tissues, organs and various tumors [11, 12]. The open reading frame of the *PD-L1* gene encodes a putative type I transmembrane protein of 290 amino acids, which consists of an extracellular region, a hydrophobic transmembrane domain and a cytoplasmic tail of 30 amino acids [13].

During T-cell activation, up-regulated PD-1 is translocated to dynamic T-cell receptor microclusters and accumulates at the signaling central supramolecular activation cluster (c-SMAC) [7]. Src homology 2 based tyrosine phosphatases (SHP-2) and SHP-1 are recruited to ITSM which locates in the cytoplasmic domain of PD-1 in the microclusters [7, 14]. Upon binding to either PD-L1 or PD-L2, recruitment of these phosphatases leads to dephosphorylation of TCR-proximal signaling molecules, including ζ-associated protein of 70 kDa (ZAP70), protein kinase C θ (PKC θ) and CD3 ζ, as well as suppressing CD28 signaling through inhibition of phosphatidylinositide 3-kinases (PI3K) and protein kinase B (Akt) activation [7, 14]. These pathways lead to suppression of the TCR and CD28 signals. Under physiological conditions, the engagement of PD-1 by PD-L1 or PD-L2 induces T cell exhaustion [15], restrains autoimmunity for maintaining immunologic tolerance and preserving physiological homeostasis [16]. However, during the phase of protective immunity against infection and tumor, the PD-1/PD-L1 engagement is regarded as a major “T cell brake” [10, 12, 17-23].

As critical regulators of T cell biology, the potent inhibitory signaling is mediated by interaction of PD-1 to PD-L1 (*K*_*d*_ of ∼8.2 µM), which is significantly weaker than that of CTLA-4/B7-1 complexes (*K*_*d*_ of ∼0.2 µM) and PD-1/PD-L2 complexes (*K*_*d*_ of ∼2.3 µM) [24, 25]. It is well known that the low affinity interaction of CD28 to B7-1 or B7-2 promotes activation of T cells, whereas the high affinity interaction of CTLA4 to B7-1 or B7-2 suppresses activation of T cells [24]. However, the resultant T cell function is not directly related to the affinity of binding B7-1 or B7-2, but to the cytoplasmic tails of CD28 or CTLA4 receptors [26-28]. Despite low affinity PD-1 and soluble PD-L1 (sPD-L1.Ig) interaction leading to suppression [29], no report describes the influence on the T cells activation due to the affinity change without alternation of the cytoplasmic tail. On the other hand, changing the PD-1 cytoplasmic tail can indeed alter the physiological function of PD-1, and lead to activation of T cells after binding to the ligand PD-L1 [30]. We are curious about the T cell status resulted from the high affinity interaction of PD-1/PD-L1.

In this study, in order to explore how T cell status was regulated by the high affinity interaction of PD-1/PD-L1, we utilized directed molecular evolution and phage-display to obtain soluble hPD-L1 variants that bound to PD-1 with enhanced affinity. We found that the interaction between PD-1 and high affinity hPD-L1 variants could modulate the inhibition of TCR-CD3 complex signal. Our study would provide evidence for manipulating the immune-check point signals of the adaptive immune response through changing the biophysical characters of the receptor/ligand interactions.

## RESULTS

### Biological activity of *in vitro* refolded hPD-1

**Figure 1:**
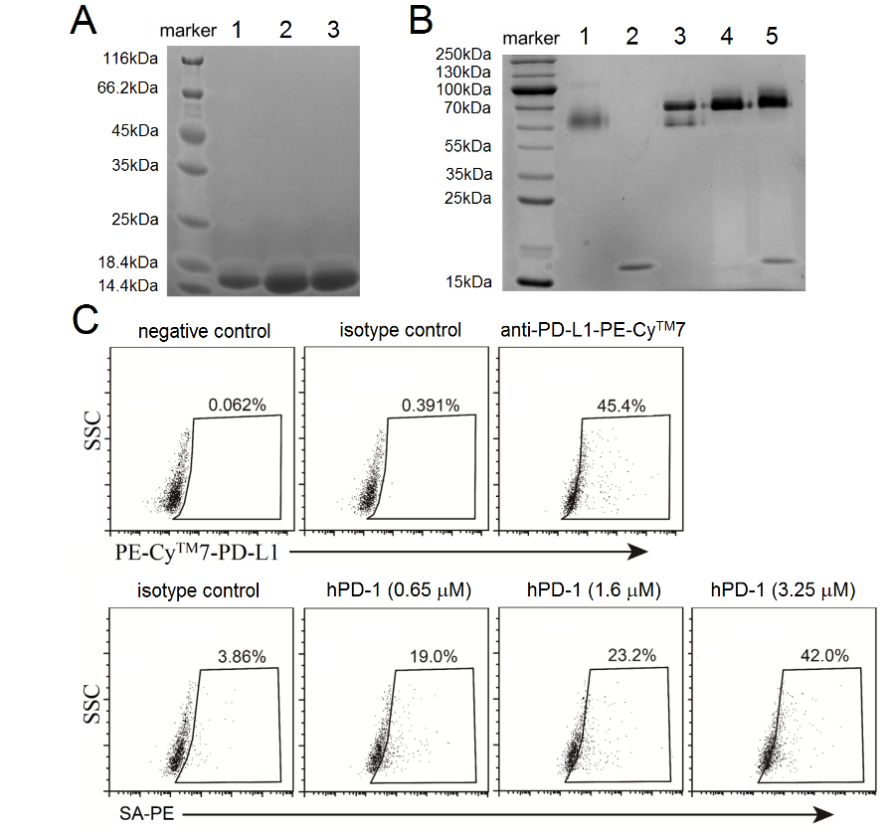
Purification of refolded and biotinylated hPD-1 and assays to confirm binding native hPD-L1 **A.** Coomassie-stained SDS-PAGE analysis of refolded hPD-1. Lane 1 to lane 3 corresponded to the bands of three fractions for single purification of hPD-1. **B.** Coomassie-stained SDS-PAGE analysis of the complex of streptavidin (SA) and biotinylated hPD-1. Lane 1: SA, 0.03 nmol; Lane 2: biotinylated hPD-1, 0.12 nmol; Lanes 3, 4 and 5 corresponded to the mixture of SA and biotinylated hPD-1 at 1:2 (0.03 nmol: 0.06 nmol), 1:4 (0.03 nmol: 0.12 nmol) and 1:8 (0.03 nmol: 0.24 nmol) molar ratios respectively. **C.** FACS analysis of biotinylated hPD-1 binding to hPD-L1 expressed on A375 tumor cells. The assays were to detect florescence generated by anti-human PD-L1-PE-Cy™7 and different concentrations (0.65 µM, 1.6 µM and 3.25 µM) of biotinylated hPD-1 followed by SA-PE respectively. The bands from coomassie-stained SDS-PAGE gel were visualized by Gel Documentation System (Bio-rad, Hercules, CA, USA) and processed by Image Lab™ software.

After dialyzing, refolded hPD-1 biotin fusion was purified by loading the dialysate to anion QHP column followed by elusion with sodium gradient. Non-reducing SDS-PAGE analysis revealed a single band, and the protein was collected at the conductivity of 0.55 mS/cm to 3.98 mS/cm (data not shown), and Figure 1A showed the representative bands from lane 1 to lane 3. Those bands migrated with an apparent molecular weight slightly higher than 14.4 KDa in the indicated lanes (Figure 1A), which was consistent with MW15.6 KDa of the protein encoded by our DNA construction. The single band indicated no misfolded aggregate of hPD-1. The hPD-1 protein refolding efficiency achieved ∼15% by using the inclusion bodies, and the purity of the protein was greater than 95% after ion-exchange, which was judged by coomassie-stained SDS-PAGE gel.

Biotinylated hPD-1 was produced with biotin and BirA biotin ligase, and purified with gel-filtration column. The void volume time of the single band of biotinylated hPD-1 was at 18 min from the column (data not shown). Biotinylated hPD-1 could be separated from BirA biotin ligase by gel-filtration chromatography and no impurity was detected in a coomassie-stained SDS-PAGE gel (Figure 1B). Non-reducing SDS-PAGE analysis revealed hPD-1 fused with biotin tag was completely biotinylated (Figure 1B).

In order to determine whether refolded biotinylated hPD-1 was able to interact with native hPD-L1, we carried out binding assays with hPD-L1 expressed on the surface of A375 tumor cells. Flow cytometry analysis showed that ∼45% of A375 tumor cells were positive and used as a control, which was stained by PE-Cy™7-conjugated anti-human PD-L1 mAb. Despite the low affinity of hPD-1 and hPD-L1 interaction [24], the fluorescence signals from the SA-PE conjugated biotinylated hPD-1 could be detected with BD Accuri™ C6 as a dose-dependent manner. The biotinylated hPD-1 at concentration of 3.25 µM could capture ∼42% of hPD-L1 positive cells, which was close to the level detected with 2.7×10^-2^ µM of the positive control antibody (Figure 1C).

These results manifested that the hPD-1 protein refolded from inclusion body *in vitro* was able to bind hPD-L1 expressed on the A375 tumor cell surface. In addition, refolded biotinylated hPD-1 could serve as a useful reagent for selection of high affinity hPD-L1 variants from the phage display libraries.

### High affinity hPD-L1 variants generated by directed molecular evolution

**Figure 2:**
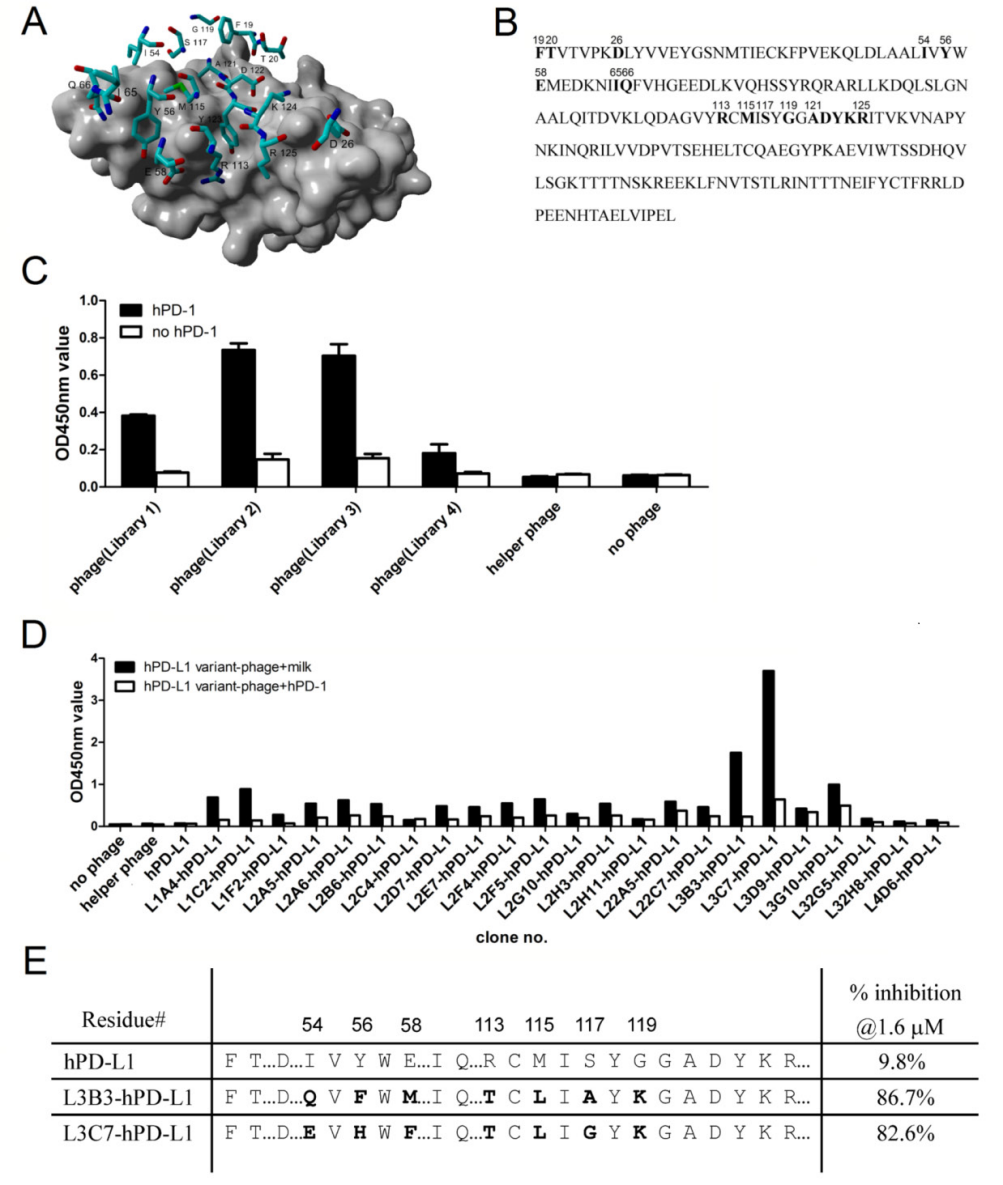
High affinity hPD-L1 variants generated by directed molecular evolution **A.** Selected residues for the mutant library construction of hPD-L1. Residues of hPD-L1 were shown as stick mode, while hPD-1 was represented by gray surface. The interface residues of hPD-L1 were chosen for constructing the mutant library. **B.** The schematic diagram of 19-229 amino acid sequence of extracellular region of hPD-L1. The targeted residues for mutation were shown in bold and the position at the original sequence was numbered. **C.** Polyclonal phage ELISA was carried out to determine the bio-panning outcomes for the four subsidiary hPD-L1 libraries. **D.** Inhibition phage ELISAs, which were setup by mixing the hPD-L1 phage variants with or without soluble hPD-1, were carried out for ranking the binding strength of positive clones selected from libraries. We tested 23 clones from 4 libraries, and native hPD-L1 phage was used as base line control, and no phage mixture and helper phage mixture were used as system negative controls. The concentration of soluble hPD-1 in the mixture was 1.6 µM. **E.** Sequences and soluble hPD-1 inhibition rates of clones L3B3-hPD-L1 and L3C7-hPD-L1. The mutated residues were shown in bold and the position at the original sequence was numbered.

In order to optimize the hPD-L1 binding to hPD-1 (*K*_*d*_ of 8.2 µM) [24], we randomized hPD-L1 residues in contact with hPD-1 deduced from structural models, and isolated high affinity hPD-L1 variants with phage display. Figure 2A showed the model which was built by YASARA program (version: 16.7.22; YASARA Biosciences GmbH, Wien, Austria) [31]. Using the crystal structure of murine PD-1 and human PD-L1 complex [32] as the reference, we are able to identify 17 residues as potential targets for improving the affinity of hPD-L1/hPD-1 interaction. Primers comprising NNK degenerate codons were used to randomize residues of F19, T20, D26, I54, Y56, E58, I65, Q66, R113, M115, S117, G119, A121, D122, Y123, K124 and R125 (Figure 2B). After the library construct being electroporated into TG1 cells, we obtained 4 libraries (L1-L4) containing estimated 2.8×10^8^ unique oligonucleotide sequences (data not shown), which were about 3-fold of the theoretical library diversity. After three rounds of bio-panning, polyclonal phage ELISA showed multiple clones having high readings, which indicated that the selection was successful, and L2 and L3 appeared to have higher signals than that of L1 and L4 (Figure 2C). We sequenced 106 clones that showed significant higher signals than that of hPD-L1 in a monoclonal phage ELISA, and 23 unique oligonucleotide sequences were found (data not shown). We used inhibition phage ELISA [33] with soluble hPD-1 to rank the binding strength of hPD-L1 phage variants showing unique sequences. The binding of hPD-L1 variant-phage to immobilized hPD-1 was well inhibited by the soluble hPD-1 in the solution, and L3B3-hPD-L1 and L3C7-hPD-L1 phage clones from library 3 (L3) demonstrated the best inhibition rates of 86.7% and 82.6% respectively (Figure 2D-2E). Sequencing analyzing indicated that there were seven residues mutated in the sequences of L3B3-hPD-L1 and L3C7-hPD-L1 respectively (Figure 2E).

### Refolding and purification of hPD-L1, L3B3-hPD-L1 and L3C7-hPD-L1

**Figure 3:**
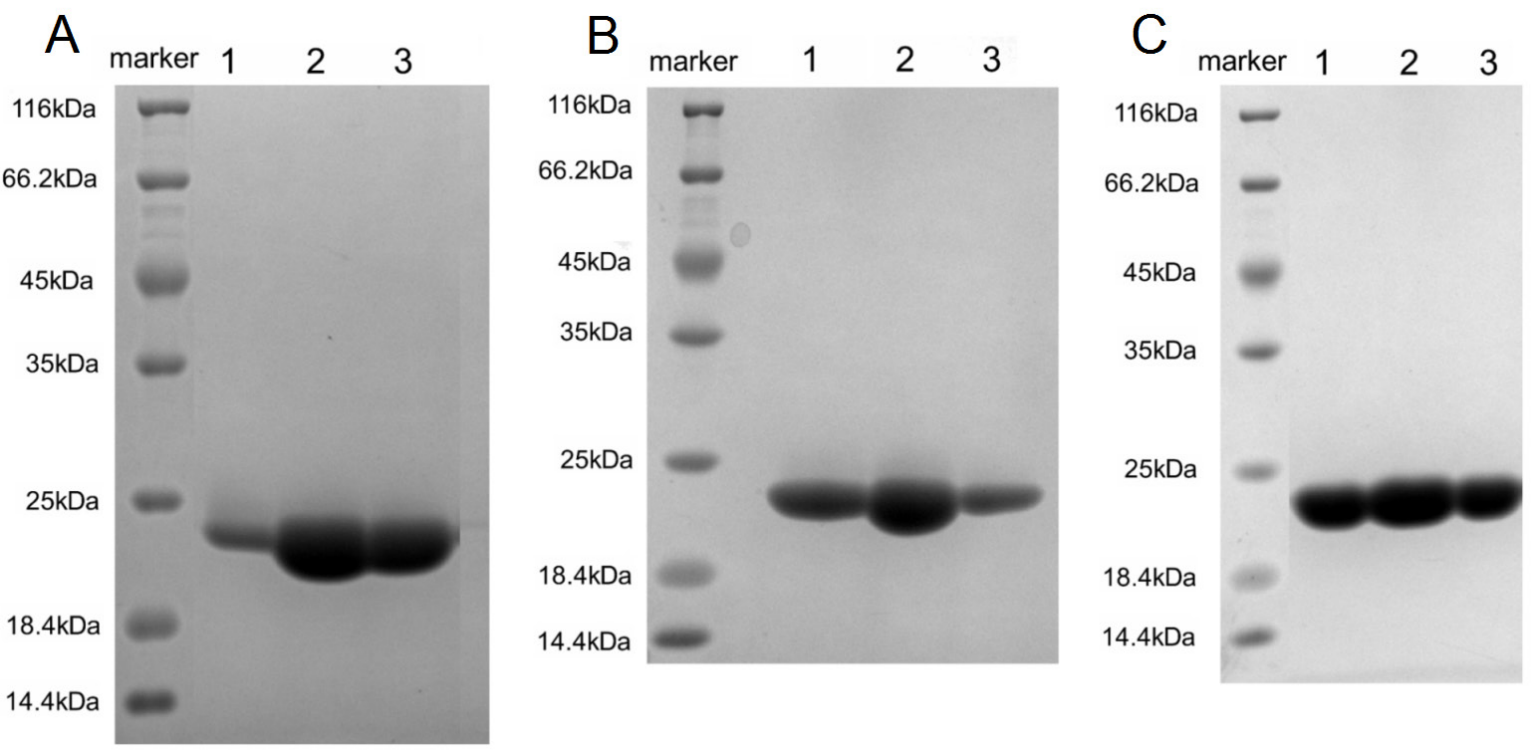
Purification of refolded hPD-L1, L3B3-hPD-L1 and L3C7-hPD-L1 **A.** Coomassie-stained SDS-PAGE analysis of refolded hPD-L1. Lane 1 to lane 3 corresponded to the bands of three fractions for single purification of hPD-L1 from gel-filtration chromatography. **B.** Coomassie-stained SDS-PAGE analysis of refolded L3B3-hPD-L1. Lane 1 to lane 3 corresponded to the bands of three fractions for single purification of L3B3-hPD-L1 from gel-filtration chromatography. **C.** Coomassie-stained SDS-PAGE analysis of refolded L3C7-hPD-L1. Lane 1 to lane 3 corresponded to the bands of three fractions for single purification of L3C7-hPD-L1 from gel-filtration chromatography. The bands from coomassie-stained SDS-PAGE gel were visualized by Gel Documentation System (Bio-rad) and processed by Image Lab™ software.

After refolding *in vitro* for three days, the dialysates containing soluble hPD-L1, L3B3-hPD-L1 or L3C7-hPD-L1 were loaded to anion QHP column respectively. Non-reducing SDS-PAGE analysis revealed a single band of hPD-L1, L3B3-hPD-L1 and L3C7-hPD-L1 were present respectively at the third peak (data not shown). The proteins were pooled and concentrated, and further purified with gel-filtration chromatography. Non-reducing SDS-PAGE analysis revealed a single band (Figure 3A-3C). Those bands migrated with an apparent molecular weight between 25 KDa and 18.4 KDa in the indicated lanes (Figure 3A-3C), which was consistent with the prediction by the DNA sequences. The inclusion bodies of hPD-L1, L3B3-hPD-L1 and L3C7-hPD-L1 refolded with ∼20% efficiency to produce proteins, and the purity was > 95% after ion-exchange chromatography and gel-filtration chromatography, which was judged by coomassie-stained SDS-PAGE gel. The purity of hPD-L1, L3B3-hPD-L1 and L3C7-hPD-L1 satisfied the requirement of Biacore assays to analyze the affinity and kinetic of hPD-L1, L3B3-hPD-L1 and L3C7-hPD-L1 to hPD-1 respectively.

### Affinity and kinetic measurements

The soluble monomers of hPD-L1, L3B3-hPD-L1, L3C7-hPD-L1 and hPD-1 were produced through inclusion bodies *in vitro* refolding and purification, and hPD-1/hPD-L1, L3B3-hPD-L1, L3C7-hPD-L1 interactions were measured by BIAcore surface plasmon resonance (SPR) assays at room temperature. For equilibrium analysis of affinity, increasing amounts of hPD-L1, L3B3-hPD-L1 and L3C7-hPD-L1 were injected respectively over immobilized biotinylated hPD-1. During the association phase, the bindings to hPD-1 of hPD-L1, L3B3-hPD-L1 and L3C7-hPD-L1 all reached equilibrium quickly. However, in the disassociation phase, hPD-L1 had a quicker off-rate in comparison with that of L3B3-hPD-L1 and L3C7-hPD-L1, and the kinetics of L3B3-hPD-L1 and L3C7-hPD-L1 were similar. The *K*_*d*_ values of 0.4994 µM, 0.75 µM and 17.66 µM were obtained for the binding of L3B3-hPD-L1, L3C7-hPD-L1 and hPD-L1 to hPD-1 respectively using a 1:1 binding model. The hPD-L1 variants L3B3-hPD-L1 and L3C7-hPD-L1 had ∼35 and ∼24 folds greater affinity for binding to hPD-1 in comparison to the hPD-L1/hPD-1 interaction (Table 2). The data of binding affinity obtained here was lower than that of the data published previously as 7.5 µM for the binding between hPD-L1 to hPD-1 [24], in which the bivalent molecule of hPD-1-Fc was used for the affinity determination.

**Table 1:** The primers of constructing hPD-L1 libraries.

Primer names	the sequences of primer (5’→3’)
PDL1-L1-YW1	GAGATGGCGCCCAACAGTCCCC
PDL1-L1-YW2	TTTCGGAACCGTAACMNNMNNTGCCATGGTATATCTC
PDL1-L1-YW3	GTTACGGTTCCGAAANNKCTGTATGTGGTTGA
PDL1-L1-YW4	ATAGACACCGGCGTCTTGCAGTTTAACATCC
PDL1-L1-YW5	GCAAGACGCCGGTGTCTATNNKTGCNNKATTTCTTATGGCGGTGCA
PDL1-L1-YW6	AAGGAAAAAAGCGGCCGCCAGTTCCGGGATAACCAGTTC
PDL1-L2-YW2	GATGTTTTTGTCTTCCATMNNCCAMNNCACMNNCAGGGCCGCCAGATCCAG
PDL1-L2-YW3	ATGGAAGACAAAAACATCNNKNNKTTCGTGCATGGCGAAGAA
PDL1-L3-YW2	ACGTTTGTAGTCTGCACCMNNATAMNNAATMNNGCAMNNATAGACACCGGCGTCTTG
PDL1-L3-YW3	GGTGCAGACTACAAACGTATCACCGTCAAAGTG
PDL1-L4-YW2	ACCGCCATAAGAAATCATGCAGCGATAGACACCG
PDL1-L4-YW3	ATGATTTCTTATGGCGGTNNKNNKNNKNNKNNKATCACCGTCAAAGTGAACGC

**Table 2:** Summary of affinity of hPD-L1 variants.

Clone no.	*k*_on_ (1/Ms)	*k*_off_ (1/s)	*K*_*d*_ (M)	T_1/2_
hPD-L1	4.361E+04	7.702E-01	1.766E-05	0.9s
L3B3-hPD-L1	7.885E+05	3.938E-02	4.994E-07	17.6s
L3C7-hPD-L1	5.192E+04	3.894E-02	7.500E-07	17.8s

*K*_*d*_ was obtained using Biacore SPR with soluble versions of hPD-L1 variants and hPD-1 immobilized by a biotin tag to a streptavidin-coated CM-5 chip surface. *k*_on_: association kinetics; *k*_off_: dissociation kinetics; *K*_*d*_​ was a ratio of *k*_off_/*k*_on_; T_1/2_: retention half-life.

### L3B3-hPD-L1 and L3C7-hPD-L1 could compete with anti-PD-1 antibody to recognize the native hPD-1

**Figure 4:**
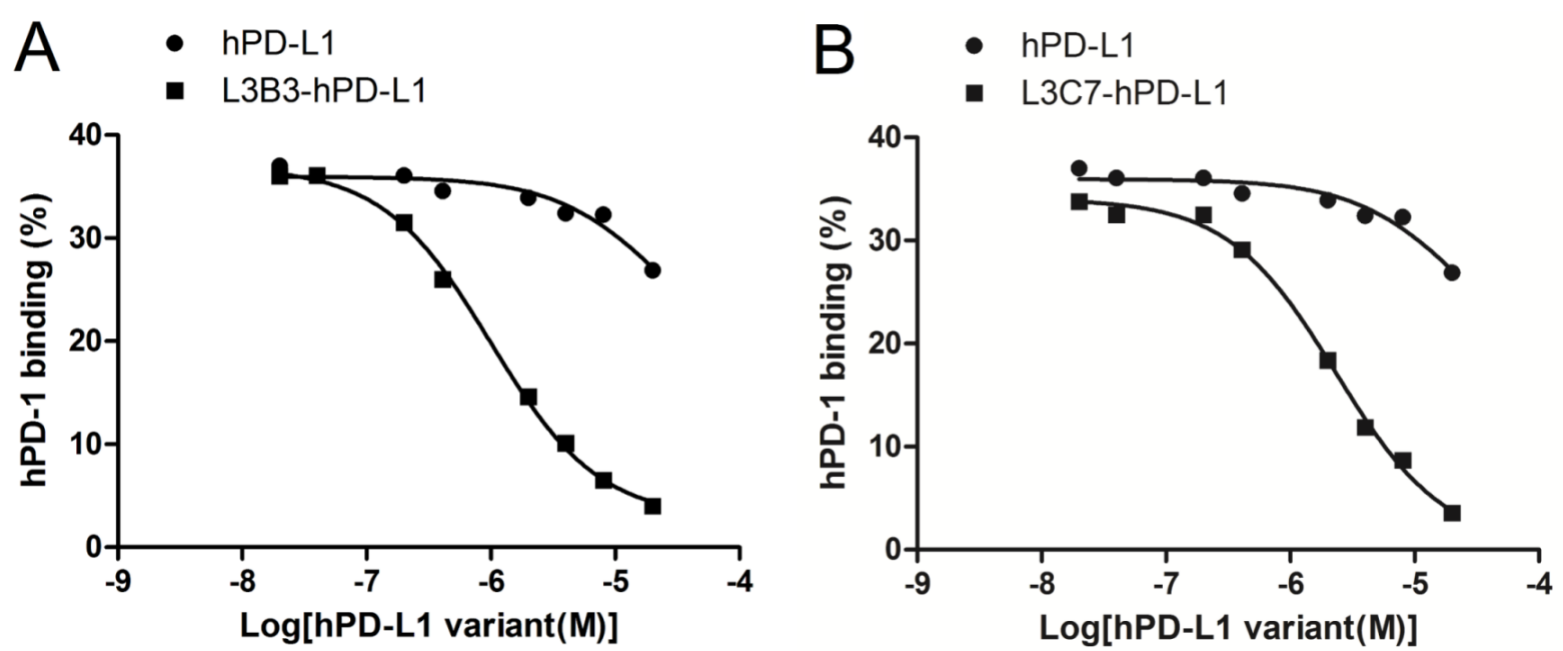
The competition hPD-1 binding assays of hPD-L1, L3B3-hPD-L1 and L3C7-hPD-L1 **A.** The hPD-L1 and L3B3-hPD-L1 competed respectively against the binding of the anti-PD-1 antibody (EH12.1). **B.** The hPD-L1 and L3C7-hPD-L1 competed respectively against the binding of anti-PD-1 antibody (EH12.1). A total of 5 µl anti-hPD-1-PE was used for each assay.

In order to assess whether hPD-L1, L3B3-hPD-L1 and L3C7-hPD-L1 could recognize native hPD-1 expressed on the surface of T cells, we performed competitive binding experiments on human PBMC, in which hPD-L1, L3B3-hPD-L1 and L3C7-hPD-L1 competed with an anti-hPD-1 antibody (EH12.1) to bind hPD-1 on the cells. Our results showed that the affinity enhanced hPD-L1 variants L3B3-hPD-L1 and L3C7-hPD-L1 could inhibit the anti-hPD-1 antibody for binding to hPD-1 in a dose dependent manner, and the IC_50_ was 1 µM (Figure 4A) and 2.3 µM (Figure 4B) respectively. Unsurprisingly, only very high concentration (20 µM) of hPD-L1 could slightly compete the binding of the anti-hPD-1 antibody with an IC_50_ of 25 µM (Figure 4A-4B). These results indicated that *in vitro* refolded hPD-L1, L3B3-hPD-L1 and L3C7-hPD-L1 could recognize the native hPD-1 at a site overlapping with that of antibody EH12.1.

### The suppressive proliferation of hPD-1 signal was attenuated by interacting with affinity enhanced hPD-L1 variants L3B3-hPD-L1 and L3C7-hPD-L1

**Figure 5:**
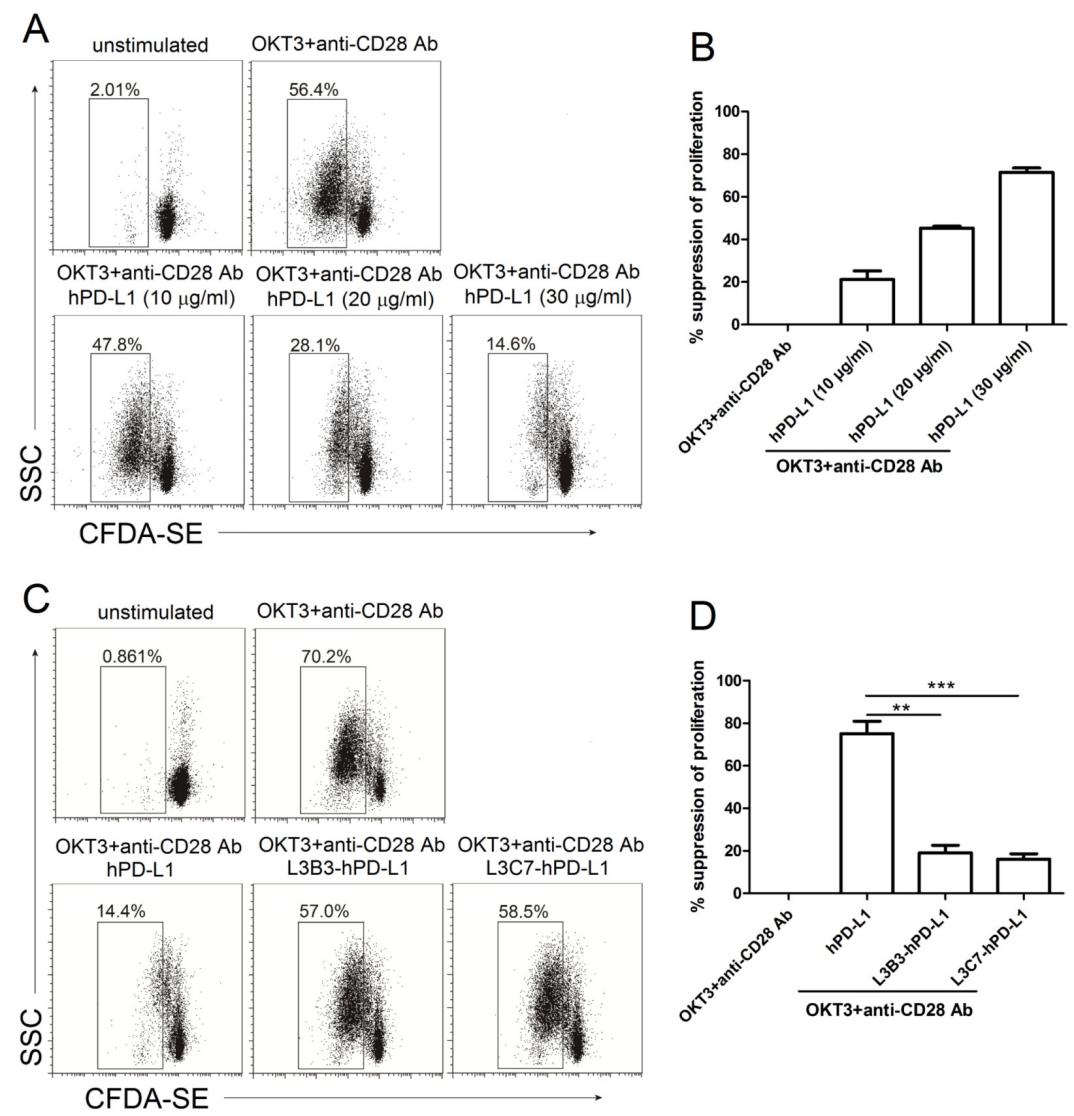
The inhibited proliferation of high affinity hPD-L1 variants/hPD-1 interaction on CD3-TCR activation was decreased **A.** The representative FACS data of proliferation of PBMC pre-stained by CFDA-SE and activated with anti-CD3 (OKT3) and anti-CD28 antibodies in the presence of different concentrations of hPD-L1. **B.** The statistical data of percent suppression of T-cell proliferation from **A.** was calculated as described in Methods and averaged from three independent experiments. Error bars indicate SEM (*n* = 3). **C.** The representative FACS data of proliferation of PBMC pre-stained by CFDA-SE and activated with anti-CD3 (OKT3) and anti-CD28 antibodies in the presence of hPD-L1, L3B3-hPD-L1 and L3C7-hPD-L1. **D.** The statistical data of percent suppression of T-cell proliferation from **C.** was calculated as described in Methods and averaged from three independent experiments. Error bars indicate SEM (*n* = 3). Unpaired student’s *t*-test, **, *P* < 0.01; ***, *P* < 0.001.

It has been reported that the interaction between PD-L1.Ig and PD-1 inhibits anti-CD3-mediated T cell proliferation [16]. In order to examine the effect of monomeric hPD-L1, L3B3-hPD-L1 and L3C7-hPD-L1 on T cell function, first, we examined the proliferation of CFDA-SE pre-stained PBMC, which were stimulated optimally with anti-CD3 and anti-CD28 antibodies in the presence of different concentrations (10 µg/ml, 20 µg/ml and 30 µg/ml) of monomeric hPD-L1. As shown in Figure 5A, monomeric hPD-L1 significantly inhibited the proliferation of PBMC induced by the antibodies in an hPD-L1 dose dependent manner. The suppressive rates of proliferation were 21.2% (10 µg/ml of hPD-L1), 45.3% (20 µg/ml of hPD-L1) and 71.5% (30 µg/ml of hPD-L1) (Figure 5B). Then, we compared the effect generated by 30 µg/ml of hPD-L1, L3B3-hPD-L1 or L3C7-hPD-L1 on the proliferation of PBMC stimulated with the anti-CD3 and anti-CD28 antibodies. As shown in Figure 5C, the high affinity hPD-L1 variants L3B3-hPD-L1 and L3C7-hPD-L1 had significantly different effects on inhibiting the proliferation of PBMC in comparison with that of soluble hPD-L1, which inhibited the proliferation of PBMC in responding to the stimulant of CD3-TCR signals with the rate of 75.1% (Figure 5D). To our surprise, the inhibition generated by the variants of L3B3-hPD-L1 and L3C7-hPD-L1 was significantly decreased in comparison to that of hPD-L1, and the inhibition rates were 19% and 16.1% respectively (Figure 5D). According to these results, the strength sequence of inhibited proliferation by soluble PD-1 ligands from strong to weak should be hPD-L1, L3B3-hPD-L1 and L3C7-hPD-L1.

### The suppressive IFN-γ secretion of hPD-1 signal was attenuated by interacting with L3B3-hPD-L1 and L3C7-hPD-L1

**Figure 6:**
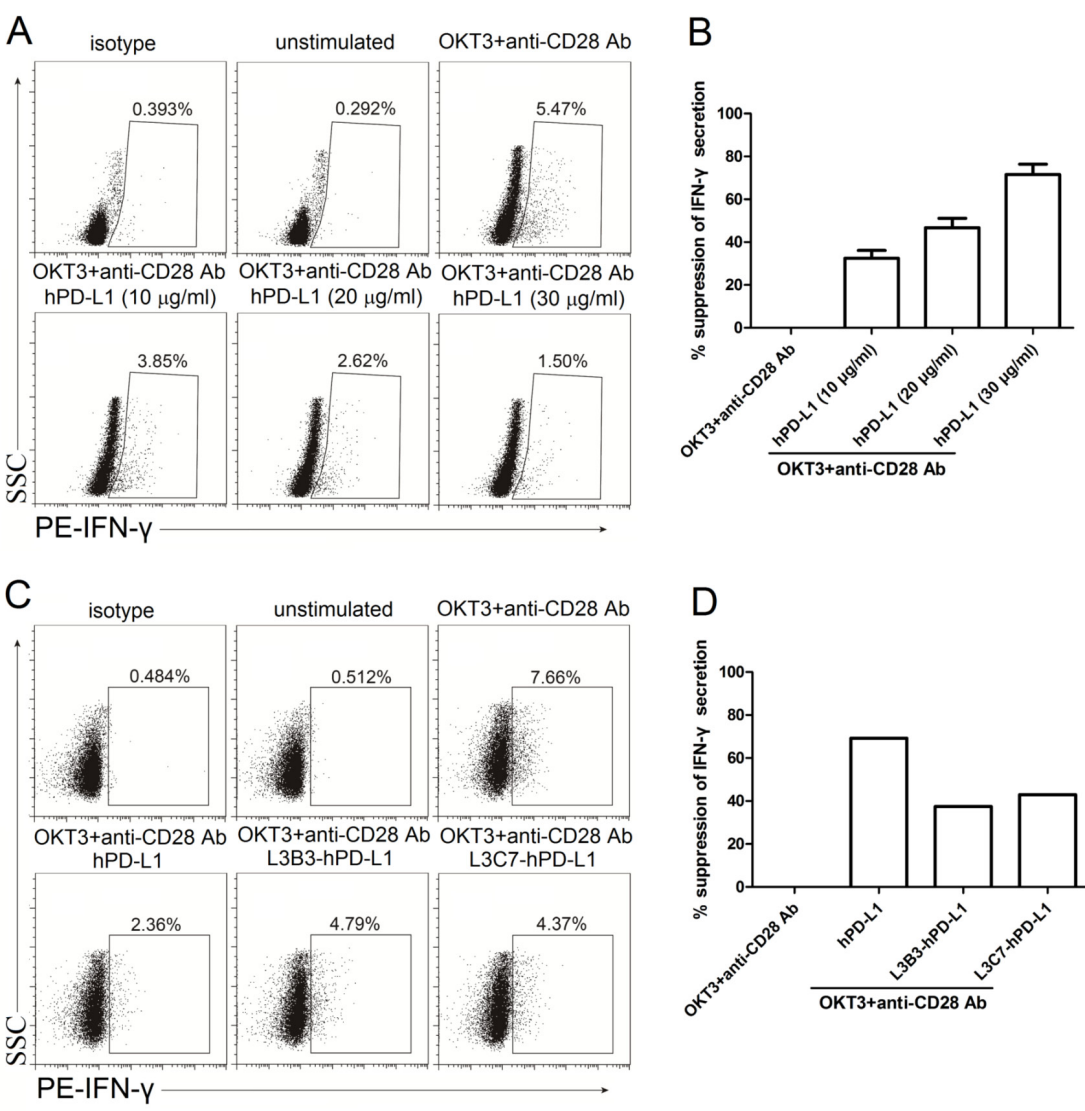
The inhibited IFN-γ secretion of high affinity hPD-L1 variants/hPD-1 interaction was decreased **A.** The representative FACS data of IFN-γ secretion of PBMC activated with anti-CD3 (OKT3) and anti-CD28 antibodies in the presence of different concentrations of hPD-L1. **B.** The statistical data of percent suppression of IFN-γ secretion from **A.** was calculated as described in Methods and averaged from three independent experiments. Error bars indicate SEM (*n* = 3). **C.** The representative FACS data of IFN-γ secretion of PBMC activated with anti-CD3 (OKT3) and anti-CD28 antibodies in the presence of hPD-L1, L3B3-hPD-L1 and L3C7-hPD-L1. **D.** Percent suppression of IFN-γ secretion from **C.** was calculated as described in Methods.

It is well established that PD-1 axis can suppress activated T cell to secrete IFN-γ [16]. The effect of L3B3-hPD-L1 and L3C7-hPD-L1 on PD-1 axis was assessed for inhibiting the secretion of IFN-γ in comparison to that of hPD-L1. In order to determine the concentration to use for the comparison, PBMC were activated with the anti-CD3 and anti-CD28 antibodies in the presence of different hPD-L1 concentrations (10 µg/ml, 20 µg/ml and 30 µg/ml), and the secretion of IFN-γ was assessed through intracellular staining followed by flow cytometry at 48 h time-points after stimulation. As shown in Figure 6A, the binding of soluble hPD-L1 significantly inhibited PBMC to secrete IFN-γ after the stimulation of anti-CD3 and anti-CD28 antibodies in a dose dependent manner. The suppressive rates of IFN-γ secretion were 32.5% (10 µg/ml of hPD-L1), 46.7% (20 µg/ml of hPD-L1) and 71.5% (30 µg/ml of hPD-L1) (Figure 6B). For comparison of suppressive effects produced by the binding of hPD-L1, L3B3-hPD-L1 and L3C7-hPD-L1 at the concentration of 30 µg/ml, the secretion of IFN-γ was analyzed in the PBMC stimulated by anti-CD3 and anti-CD28 antibodies. The IFN-γ secretion inhibited by high affinity variants L3B3-hPD-L1 and L3C7-hPD-L1 was attenuated in comparison with that by hPD-L1 (Figure 6C), and the inhibition rates were 37.5% and 43% respectively for the two variants (Figure 6D), which had the same trend shown by the proliferation experiments.

## DISCUSSION

The activation and proliferation of T cells are necessary components of effective acquired immune responses. The T cell activation efficiency needs proper regulatory measures to inhibit immune hyper-activation, which leads to autoimmune diseases. The regulation of T cell activation and proliferation relate to the coordinated interaction of multiple signaling pathways. The PD-1 and PD-L1 interaction of PD-1 axis negatively regulates the T cell responses and maintains tolerance to self-antigens and limits inappropriate immune activities with much low affinity (*K*_*d*_ of ∼8.2 µM) in comparison to that of negative regulator CTLA-4 and B7-1 (*K*_*d*_ of ∼0.2 µM). On the other hand, the inhibition pathway of PD-1 provides opportunities to develop strategies to evade host immune surveillance for cancer and chronic infections.

The crystal structure of murine PD-1 and human PD-L1 complex (PDB: 3SBW) revealed the contact interface involving total 14 residues of hPD-L1 [32]. There is 64% protein sequence identity between murine and human PD-1, which can cross interact with PD-L1 of both species with similar affinities [9, 16, 34]. With this information, we used YASARA program to create a molecular model for the structure of hPD-L1/hPD-1 complex, which indicated that additional 3 residues of E58, I65 and G119 also involved in the interface of hPD-L1 to hPD-1. We selected 17 residues of hPD-L1 to build mutation libraries for improving the affinity, which covered the complete 13 contact residues identified with recently available hPD-1/hPD-L1 complex structure (PDB: 4ZQK). The hPD-1/hPD-L1 structure revealed that 12 out of the 13 contact residues were identical as the 14 contact residues of mPD-1/hPD-L1 complex [35]. We found that there were three residues T20, E58 and S117 of hPD-L1 contributed differently in two complexes of mPD-1/hPD-L1 and hPD-1/hPD-L1, while the T20 and S117 only played contacting roles in the former complex, the E58 only in the latter one. The isolated high affinity variants L3B3-hPD-L1 and L3C7-hPD-L1 had mutations of E58M, S117A and E58F, S117G respectively, which demonstrated potential structural significance in the interaction of hPD-L1 and hPD-1. These results showed that the point-mutation in this study covered all contact residues of hPD-L1 to hPD-1 for the optimization of binding affinity.

The biological functions of PD-L1 have been analyzed *in vivo* and *in vitro* previously, and we are trying to analyze the potential change of the biological function of high affinity hPD-L1 variants for suppressing T cell activation. Previous studies have demonstrated that PD-1 deficient mice developed autoimmune diseases [36], and PD-1 axis blockage rescued the tumor clearance capability of T cells [10, 18, 20]. It has been established that high levels of detected soluble PD-L1 in peripheral blood indicated poor cancer prognosis [37]. These *in vivo* facts confirm the role of PD-1 axis in immune suppression. However, because different *in vitro* conditions used, the role of PD-L1 in the regulation of T cell responses seems to be complicated and is still under debate. Hideto Tamura et al. reported mPD-L1.Ig promoted the proliferation of T cells stimulated with a suboptimal dose of anti-mouse CD3 antibody, whilst both reagents were pre-coated on the 96 well plates [11]. Gordon J. Freeman et al. reported that the proliferation of murine anti-CD3/anti-CD28 antibody activated murine T cells was inhibited with immobilized hPD-L1.Ig on plastic surface or beads [16]. Davide Brusa et al. reported the PD-L1-Fc chimeric protein significantly decreased the secretion of IL-4 and IFN-γ by anti-CD3/CD28 mAbs stimulated T cells from chronic lymphocytic leukemia (CLL) patients [29]. Haidong Dong et al. reported soluble PD-L1.Ig in the cultures mostly decreased its co-stimulatory effect [13]. The different functions of the interaction of PD-1 and PD-L1 may depend on antigen-TCR binding strength of the target cell, or there is possible an additional counter-receptor for PD-L1 except PD-1 on T cells [11]. However, under identical anti-CD3/CD28-mAb stimulation conditions, we found that immobilized hPD-L1 promoted PBMC to secrete IFN-γ, but soluble hPD-L1 did the opposite effects to inhibit the cytokine secretion (data not shown). Therefore, the *in vitro* effect of the soluble hPD-L1 binding to T cell surface PD-1 conforms to the *in vivo* fashion of PD-1-axis function. In contrast our results demonstrated that L3B3-hPD-L1 and L3C7-hPD-L1 significantly attenuated the inhibition of PD-1 axis to the proliferation and IFN-γ secretion of PBMC.

Considering potential molecular mechanism of the reduced inhibition, we find that the cytoplasmic tail of CD150 has a paired of tyrosine-based motifs (TxYxxV/I), which bind to SH2-containing inositol phosphatase (SHIP) depended on the presence of the small adapter SH2D1A (SH2-domain-containing gene 7A), otherwise it will bind to SHP-2. The motif (TxYxxV/I) was designated as ITSM [38]. The previous study indicated gene mutation of *SH2D1A*, which was thought to act as a negative regulator of immune cell activation, leaded to X-linked lymphoproliferative syndrome (XLP) [39], in contrast, SHP-2 acted in positive manner to transduce signals from receptor protein tyrosine kinases [14]. This was proposed as a mechanism by which CD150 could have both positive and negative effects concerning B cell activation [38]. The cytoplasmic tail of PD-1 also contains ITSM, but the ITSM of PD-1 shows a different function from the ITSM of CD150 when ligating to PD-L1 with low affinity. Both a murine B cell tumor line model [40] and a primary human CD4^+^ T cell model [14] suggested that SHP-2 was a key downstream inhibiting signaling molecule that recruited to the ITSM of PD-1, instead, the ITSM of PD-1 failed to recruit SH2D1A [14]. In order to unveil the mechanism of the high affinity ligation of hPD-L1 variants with PD-1 decreasing the inhibitive function for activated T cells, we will have to study whether it involves in the decrease of SHP-2 recruiting to ITSM of PD-1, or the switch of SHP-2 and SHIP by the ITSM.

On the other hand, it is intriguing that the binding of high affinity PD-L1 variants L3B3-hPD-L1 or L3C7-hPD-L1 to PD-1 on T cells leads to less inhibition of T cell activation in comparison with that of low affinity hPD-L1. PD-L1/PD-1 interaction initiates PD-1 suppressive effects, and sustained interaction generated by high affinity hPD-L1 may be expected to produce strong PD-1 stimulation and enhance the suppressive effects. During our investigation of this surprise result, it was reported that PD-1 axis mediated T cell suppressive potency was determined by the strength of PD-1 signaling [41]. The T cell surface PD-1 expression levels could define the strength of PD-1 axis to suppress CD3-TCR signals [41]. These authors demonstrated that high levels of PD-1 expression increased the number of PD-L1 to PD-1 ligation and produced stronger suppressive PD-1 signals than that of low levels of PD-1 expression [41]. In our study, we showed that the binding of soluble hPD-L1 to the PD-1 on T cell surface inhibited the proliferation and IFN-γ secretion of PBMC in a dose dependent manner (Figure 5A and Figure 6A). The low affinity interaction of hPD-L1/hPD-1 had *K*_*d*_ of 17.66 µM, which was resulted mainly from a quick off-rate (Table 2). This means that a high concentration of PD-L1 should lead to high frequent PD-1/PD-L1 on/off, which will cause high frequent stimulation of ITSM of the PD1. The resultant actions will suppress CD3-TCR signals. At the condition of previous study [41], the increased ligation of PD-L1 to PD-1 could be converted by increased on/off frequency of PD-L1/PD-1, and the levels of this frequency determined the strength of PD-1 signals. The high affinity variants L3B3-hPD-L1 or L3C7-hPD-L1 have much slow off-rates than that of hPD-L1, which indicates lower on/off frequencies for PD-1/L3B3-hPD-L1 or L3C7-hPD-L1 than that of hPD-1/hPD-L1 interaction. As a result, high affinity variants L3B3-hPD-L1 or L3C7-hPD-L1 should attenuate the suppressive strength of PD-1 signals.

In conclusion, it is well documented that the concentration of soluble PD-L1 in patient peripheral blood negatively correlates with the cancer prognosis, the role of the soluble PD-L1 in cancer progress remains to be revealed with further investigation. However, it is the first time to show that ligation of hPD-1 with high affinity soluble hPD-L1 variants can attenuate the suppressive function of the T cells. We speculate high affinity hPD-L1 variants optimized further may block PD-1 axis, and believe that our results can provide a new strategy for manipulating the regulation of activated T cells. In addition, high affinity hPD-L1 may also be developed as a kind of new biologics for reversing immune-suppressive tumor micro-environment and treating cancer.

## MATERIALS AND METHODS

### Construction, expression, refolding and purification of human PD-1 (hPD-1) with biotin tag, soluble human PD-L1 (hPD-L1) and hPD-L1 variants (L3B3-hPD-L1 and L3C7-hPD-L1)

Cloning primers and synthetic genes encoding 33-150 residues of hPD-1 (NCBI: NM_005018.2) and 19-229 residues of human PD-L1 (GenBank: AY254342.1) were ordered from GenScript (Nanjing, China) for the expression in *E. coli*. The hPD-1 residue 93 Cys was mutated to Ser to remove a free thiol from the bacterial expressed protein for improving refolding. The PCR products of hPD-1 and hPD-L1 genes were digested with Nco I and Not I endonuclease (NEB, Ipswich, MA, USA), and ligated into the pET28a vector (Novagen, Madison, WI, USA) which had a biotin tag gene for encoding tagged proteins and predigested by the identical restriction enzymes. The genes of hPD-L1 variants obtained from hPD-L1 phage libraries were amplified by PCR from the pGZ196 vector (in house construct), and cloned into the pET28a vector. The ligated products were transformed routinely into the competent *E. coli* strain BL21 (DE3) (Vazyme, Nanjing, China). Successful clones were confirmed with DNA sequencing.

Transformed cells containing pET28a constructs were grown in Luria-Bertani (LB) medium supplemented with 50 µg/ml kanamycin overnight at 37 °C, and the proteins were expressed as inclusion bodies by induction for 4 h with 1 mM isopropylthiogalactosidase (IPTG) at the next day. The collection of cells and purification of inclusion bodies were executed as previously described [42]. The purified inclusion bodies were solubilized in guanidine buffer, and the procedures of refolding proteins were completed as published previously [24].

After filtration through a 0.45 µm membrane, the dialysis mixture was loaded to an anion QHP column (GE Healthcare, Chicago, IL, USA); bound proteins were eluted by sodium gradients of achieving 100% 1 M NaCl in 80 min produced with buffer of 10 mM Tris-HCl and 1 M NaCl, and collected and checked with SDS-PAGE at each peak. Refolded proteins were pooled and concentrated by ultrafiltration. For hPD-L1 and hPD-L1 variants, proteins were further loaded to Superdex™ 75 10/300GL gel filtration column (GE Healthcare) with PBS at pH 7.2; hPD-1 protein samples containing biotin tag were buffer exchanged with 10 mM Tris-HCl pH 8.0 for further process as following methods.

### Biotinylation of hPD-1 with biotin tag

The hPD-1 protein with biotin tag was biotinylated with a kit from Avidity (Aurora, Colorado, USA) according to the manufacturer’s instructions. Briefly, the components of biotinylated reaction were hPD-1 with biotin tag as the substrate for BirA biotin ligase. The working concentration of protein substrate was adjusted to 40 µM at the final reaction mixture, which contained diluted 10-fold Biomix-A and Biomix-B, and 2.5 µg of BirA biotin ligase (per 10 nmol substrate) and incubated for 40 min at 30 °C. The completed reaction mixture was concentrated by ultrafiltration, and loaded to Superdex G-75 gel filtration column with PBS pH 7.2. The purity of biotinylated hPD-1 was determined by SDS-PAGE. The percentage of biotinylation of hPD-1 was evaluated using following methods. Streptavidin (SA) and biotinylated hPD-1 were mixed at 1:2, 1:4, and 1:8 molar ratios and incubated for 30 min at 37 °C, then the mixture was analyzed by SDS-PAGE to show the amount of the binding of biotinylated hPD-1 to SA.

### Construction of hPD-L1 libraries

Seventeen PD-1 contact residues in hPD-L1 were determined using crystal structure of the mPD-1(mouse PD-1)/hPD-L1 (human PD-L1) complex (Protein Data Bank (PDB): 3SBW). In order to investigate if there was an optimal contact, identified contact residues were randomized with degenerate codons of NNK and overlapping PCR to build libraries, which were cloned into a phage display vector pGZ196 with the primers listed in Table 1. The hPD-L1 libraries were grouped into four working libraries for selection of high affinity variants. The total theoretical diversity of unique oligonucleotide sequences of the libraries was 9.1×10^7^. The PD-L1 variant fragments were cloned to fuse at 5’-end of the M13 gene three in pGZ196 vector at *Nco* I/*Not* I sites. After ligation, the DNA were purified and concentrated with a DNA purification kit (Qiagen, Carol Stream, IL, USA) according to the manufacturer’s instructions. We electroporated 200 µl of TG1 competent cells (Lucigen, Middleton, Wisconsin, USA) with 800 ng of pGZ196: hPD-L1 constructed DNA to make libraries.

### Selection of hPD-L1 libraries

High affinity hPD-L1 variants were selected by bio-panning with the phage libraries on immobilized hPD-1 receptor. The selection methods were similar as those described previously [43]. Briefly, the biotinylated hPD-1 was captured on beads pre-coated with streptavidin, and the hPD-L1 phage display libraries were allowed to bind the immobilized hPD-1 for 1 h. Nonbinding phages were removed by sequential washing. Binding phages were then eluted from the beads by adding 500 µl trypsin (100 µg/ml) and stirred for 1 h at room temperature. The eluted phages were used to infect 5 ml suspension of TG1 *E. Coli* cells, followed by spreading on TYE plates supplemented with 100 µg/ml ampicillin and 2% glucose and growing overnight at 37 °C in an incubator.

### Screening for high affinity binders

After the last round of selection, we assessed individual ampicillin-resistant colonies as described previously [43]. Briefly, individual colonies from the titration plates were picked into 2×TY medium supplemented with 100 µg/ml ampicillin and 2% glucose in a 96-well microtiter plate, and grown shaking for 4 h at 37 °C. The culture was added with 1×10^8^ of M13 helper phages and incubated for 1 h at 37 °C. The cells were pelleted and re-suspended in 2×TY medium supplemented with 100 µg/ml ampicillin, then grown shaking overnight at 30 °C. The supernatant containing phages were obtained by spun down the cells at 1800 *g* for 10 min at 4 °C, and was used for screening high affinity binders.

To screen binders, we captured biotinylated hPD-1 on 96-well Nunc MaxiSorp plates pre-coated with streptavidin and non-specific protein binding surface was blocked with blocking buffer (PBS containing 3% skimmed milk) for 1 h. Phage supernatant pre-mixed with the blocking buffer was added to the 96-well plate at the same volume used for the biotinylated hPD-1 coated on the plate. The hPD-1 binding by phage displayed hPD-L1 variants was detected with horseradish-peroxidase (HRP)-conjugated anti-M13 antibody (1:1000 dilution; Amersham-Pharmacia, Little Chalfont, UK), and the antibody binding signals were revealed with TMB reaction buffer and read at 450 nm.

The affinities of selected binders were ranked with inhibitive phage ELISA. Briefly, the phage displaying hPD-L1 variants were incubated with 1.6 µM non-biotinylated hPD-1 for 1 h before adding to the 96-well plate pre-treated as above. Binding phage remaining in the solution was detected as above.

### Determination of hPD-L1 and hPD-1 affinities with surface plasmon resonance (SPR)

The affinity was determined by Biacore T200 (GE Healthcare). A CM5 BIAcore chip was coated with streptavidin using amine coupling, then biotinylated hPD-1 was captured on the active channel. After blocking non-specific binding surface with 50 mM biotin, both the reference and active channels were injected hPD-L1 and its variants sequentially at various concentrations, and a channel injected with buffer alone was used as a blank control. The affinities were determined with the method of single-cycle kinetics. The data was fitted with a 1:1 binding model using Biacore T200 evaluation software to obtain the kinetics constants (*k*_on_: association constant; *k*_off_: dissociation constant; *K*_*d*_ = *k*_off_/*k*_on_).

### Validate bacterial expressed hPD-1 by surface staining of A375 cells

The A375 cells were purchased from the American Type Culture Collection (ATCC, Manassas, VA, USA), and grown in Dulbecco’s modified Eagle’s medium (DMEM; Gibco, Waltham, MA, USA) supplemented with 10% heat-inactivated fetal bovine serum (FBS; Gibco), and maintained at 37 °C in a humidified 5% CO_2_ incubator. The A375 cells were incubated with anti-PD-L1-PE-Cy™7 (BD Pharmingen, San Diego, CA, USA) at room temperature for 30 min. For indirect immunofluorescence, the cells were incubated with escalated concentrations (0.65 µM, 1.6 µM and 3.25 µM) of biotinylated hPD-1 at room temperature for 30 min. The cells were washed twice with FACS buffer (PBS containing 2% FBS), and further incubated with SA-PE (BD Pharmingen) at room temperature for 30 min. SA-PE and/or anti-mouse IgG1 κ-PE-Cy™7 (BD Pharmingen) were used to stain the same number of A375 cells at the same conditions as negative controls for the flow cytometric analysis. Fluorescence was evaluated by FACS analysis using BD Accuri™ C6 within 4 h, and the data was analyzed using FlowJo 7.6 software (Tree Star, Ashland, OR, USA).

### Analysis of proliferation and interferon γ (IFN-γ) secretion by PBMC

Peripheral blood from anonymous healthy donors were obtained from the Guangzhou blood center, and peripheral blood mononuclear cells (PBMCs) were isolated by Ficoll-Hypaque gradient centrifugation.

For proliferation assays, PBMCs were pre-stained with the final concentration of 1 µM carboxyfluorescein diacetate succinimidyl ester (CFDA-SE; Molecular Probes, Eugene, OR, USA) as described previously [44], and 4×10^5^ /well pre-stained PBMCs were cultured in 96-well flat-bottomed plates in a volume of 200 µl RPMI-1640 containing 10% FBS. The PBMCs were stimulated by adding the combination of OKT3 (Biolegend, San Diego, CA, USA) and anti-CD28 Ab (R&D systems, Minneapolis, MN, USA) at the final concentrations of 50 ng/ml and 30 ng/ml respectively, and assays were carried out in the presence or absence (as a comparable control) of hPD-L1 (10 µg/ml, 20 µg/ml and 30 µg/ml), L3B3-hPD-L1 (30 µg/ml) or L3C7-hPD-L1 (30 µg/ml) respectively. The culture was incubated at 37 °C in a humidified 5% CO_2_ incubator for 6 days for duration of the whole assay. On day 3, recombinant human interleukin-2 (R&D systems) was added to a working concentration of 50 IU/ml.

For IFN-γ secretion assays, PBMCs without pre-stained were stimulated as above. The cells were added with 0.13 µl of BD GolgiStop™ per well and incubated for the last 6 hours of 48 h activation assays. Cells were collected and fixed/permeabilized with Cytofix/Cytoperm solution (BD Pharmingen) according to the manufacturer’s instructions, and stained with anti-human IFN-γ-PE (BD Pharmingen). Fluorescence was evaluated and the data was analyzed as above.

### Experiments of competitive combination

To prepare activated T cells, freshly isolated human PBMCs (2×10^6^ cells/ml) were stimulated with 10 ng/ml phorbol 12-myristate 13-acetate (PMA; Sigma-Aldrich, St Louis, MO, USA) and 1 µg/ml ionomycin (Ion.; Sigma-Aldrich) in a volume of 200 µl RPMI-1640 containing 10% FBS at 37 °C in a humidified 5% CO_2_ incubator for 24 h. Cells were collected and washed twice with FACS buffer, and added anti-human PD-1-PE (BD Pharmingen) and hPD-L1, L3B3-hPD-L1 and L3C7-hPD-L1 respectively at serial concentrations (0.02 µM, 0.04 µM , 0.2 µM , 0.411 µM , 2 µM , 4 µM , 8 µM and 20 µM) in the same time, and incubated in room temperature for 1 h. Samples added with anti-human PD-1-PE (BD Pharmingen) and anti-mouse IgG1κ-PE (BD Pharmingen) were considered as positive control and negative control respectively. Fluorescence was evaluated and the data was analyzed as above.

### Statistical analysis

Statistical analysis and graphical presentations were computed with GraphPad Prism V5.0 software (GraphPad Software Inc., San Diego, CA, USA). Percent suppression was calculated using the formula: percent T-cells responding in unsuppressed condition minus percent T-cells responding in suppressed condition divided by percent T-cells responding in unsuppressed condition×100. The data was expressed as the mean ± SEM. Unpaired student’s *t*-test was used to determine the statistical significance between groups, with *P* < 0.05 being considered significant.

## Abbreviations

TCR: T cell receptor
ZAP70: ζ-associated protein of 70 kDa
PD-1: programmed cell death protein 1
ITIM: immune receptor tyrosine-based inhibition motif
ITSM: immune receptor tyrosine-based switch motif
SHP-2: Src homology 2 based tyrosine phosphatases
PKC θ: protein kinase C θ
XLP: X-linked lymphoproliferative syndrome
SHIP: SH2-containing inositol phosphatase
SH2D1A: SH2-domain-containing gene 7A
FACS: fluorescence-activated cell sorting
Fc: crystalizable fragment
c-SMAC: central supramolecular activation cluster
PI3K: phosphatidylinositide 3-kinases
Akt: protein kinase B
